# Spectrogram Inversion for Reconstruction of Electric Currents at Industrial Frequencies: A Deep Learning Approach

**DOI:** 10.3390/s24061798

**Published:** 2024-03-11

**Authors:** Abderraouf Lalla, Andrea Albini, Paolo Di Barba, Maria Evelina Mognaschi

**Affiliations:** Department of Electrical, Computer and Biomedical Engineering, University of Pavia, Via Ferrata 5, 27100 Pavia, Italy; abderraouf.lalla01@universitadipavia.it (A.L.); andrea.albini@unipv.it (A.A.); paolo.dibarba@unipv.it (P.D.B.)

**Keywords:** magnetic field measurements, current reconstruction, spectrogram, deep learning, CNN

## Abstract

In this paper, we present a deep learning approach for identifying current intensity and frequency. The reconstruction is based on measurements of the magnetic field generated by the current flowing in a conductor. Magnetic field data are collected using a magnetic probe capable of generating a spectrogram, representing the spectrum of frequencies of the magnetic field over time. These spectrograms are saved as images characterized by color density proportional to the induction field value at a given frequency. The proposed deep learning approach utilizes a convolutional neural network (CNN) with the spectrogram image as input and the current or frequency value as output. One advantage of this approach is that current estimation is achieved contactless, using a simple magnetic field probe positioned close to the conductor.

## 1. Introduction

Smart systems have assumed a significant role in engineering, extending their influence into various daily life tasks. This transfer of intelligence from human beings to systems is facilitated by modern tools and techniques falling under the branch of artificial intelligence.

In the field of electric measurements, smart meters and procedures are becoming more and more utilized in an industrial and civil environment. For the sake of an example, Smart Electric Meters record energy-consumption data at short intervals and transmit them back to the utility provider [1,2]. They often support bidirectional communication and may have additional features, such as remote disconnect/reconnect capabilities. Submetering systems involve the installation of additional meters at specific points within a facility to monitor energy consumption in different areas or with specific equipment. Wireless Sensor Networks use wireless communication to connect sensors and devices for monitoring various parameters, including electric quantities and energy consumption [3,4]. These sensors can be deployed in both indoor and outdoor environments.

Nevertheless, one of the areas where the need to measure electric parameters is most critical is within the field of electric energy distribution, specifically in power lines. The measurement of current in power lines is indispensable for the monitoring and management of electrical systems. A reliable, cost-effective, and contactless method for current measurement in power lines could offer numerous advantages.

Firstly, contactless measurement eliminates the requirement for physical connections to the power lines, reducing the risk of accidents and ensuring the safety of personnel involved in the measurement process. Traditional current measurement methods often entail opening circuits or employing physical clamps, which can be hazardous, especially in high-voltage environments [5].

Furthermore, contactless measurements can be conducted without interrupting the power supply, resulting in minimal disruption to operations. This is particularly crucial in industries where continuous power is essential and shutting down systems for measurements is not feasible.

Contactless current measurement also allows for the remote monitoring of power lines. This is particularly beneficial in situations where accessing the location or the power lines is challenging, such as in remote areas or at great heights.

Furthermore, contactless current measurement techniques offer greater flexibility in terms of where and how measurements can be taken. This adaptability is beneficial in situations where physical access to power lines is limited or where changes in the power system configuration occur frequently.

Finally, contactless measurement systems can provide real-time data, enabling prompt responses to changes or issues in the power system. This can be valuable for optimizing system performance, preventing failures, and ensuring the overall stability of the power-distribution network [6].

In summary, the contactless measurement of current in power lines significantly enhances safety, reliability, and efficiency in power-distribution systems. It augments the capabilities of monitoring and control systems, ultimately resulting in better overall performance of the electrical grid. However, the hazards associated with the presence of both the magnetic field at an industrial frequency [7] and the electric current in the power lines can be substantially reduced when the current is accurately known through measurements.

The study also places a strong emphasis on the concept of smart grids, drawing inspiration from the vision of a technologically advanced company where the maintenance department operates in a highly automated and interconnected environment. In the context of this model, the concept of a smart grid involves leveraging advanced technologies and data-driven approaches to enhance the efficiency and intelligence of power systems.

In a smart company setting, the maintenance department is envisioned as a hub of information, utilizing advanced data analytics, machine learning, and interconnected systems. This informatization allows for real-time monitoring, predictive maintenance, and agile responses to potential issues. The interconnected nature of the maintenance processes ensures seamless communication and coordination among various components of the system.

By integrating the principles of smart grids into the model, there is a focus on optimizing energy consumption, improving reliability, and incorporating intelligent decision-making processes. The interconnectedness of systems enables a holistic approach to energy management, where data from various sources is leveraged to make informed decisions, enhance operational efficiency, and contribute to a more sustainable and resilient energy infrastructure. In essence, the study not only presents a predictive model for the current but also aligns with the broader vision of smart grids and informatized maintenance practices in modern industrial settings.

In recent years, automated maintenance has gained a prominent role in the industrial environment [8]. Specifically, the use of informatics system for the automated detection of faults has been explored in many research fields. As an example, deep neural networks, including Convolutional Neural Networks (CNNs), have been widely applied for the detection of faults in electrical machines and electric systems [9,10].

In general, the application of deep learning has proven to be successful in solving various problems in both low- and high-frequency electromagnetism [11,12]. The outcomes of these studies are encouraging. Building upon this background and these studies, the method proposed in this paper aims to take the initial steps toward establishing an automated maintenance system for power lines and smart grids.

Furthermore, our model demonstrates the rapid and accurate prediction of the current, a critical feature for industries where real-time information is crucial. The data collected during the acquisition process are organized in the form of matrices, facilitating efficient computation and storage. The model exhibits a streamlined training process, consuming minimal time to establish a well-trained network. Its flexibility is highlighted by the adaptability of the data, allowing it to be tailored for various applications.

Moreover, leveraging advanced communication protocols has the potential to significantly enhance the performance of this model. By incorporating cutting-edge communication technologies, the efficiency and responsiveness of the model can be further optimized, making it even more adept at meeting the dynamic and evolving demands of industrial applications. The combination of rapid prediction, efficient data handling, and adaptability positions this model as a valuable asset for industries seeking agile and effective solutions.

To achieve this goal, the paper focuses on measuring current in a contactless setup by utilizing the magnetic field generated by the same current. Magnetic field data are acquired using a magnetic probe, representing the spectrum of frequencies of the magnetic field over time (spectrogram). The spectrogram is visualized as an image, referred to as a “waterfall” in the subsequent discussion. Waterfalls illustrate color density corresponding to the magnetic field value and the frequency band over time. One of the key points of the work is to generate the database of images, by varying the current flowing in the conductor, in terms of amplitude and frequency. Specifically, the objective is to create a smart system capable of establishing connections between the patterns observed in the captured magnetic field waterfalls and the actual current values.

Machine learning comes in handy for this situation, by using the waterfall images to treat and transfer them to RGB matrices. RGB matrices give definition to each pixel in the image [13]. The latter will be used for training the CNN that has, as an input, the RGB matrices and the output is regression followed by the estimation of the current (either amplitude or frequency). The algorithm learns from the provided RGB matrices of the database and, in the future, will be able, if properly trained, to project the learning to collected images from new measurements, in order to estimate the current.

The study encounters various challenges, particularly the requirement for a high number of images (measurements) to effectively train the Convolutional Neural Network (CNN). To assess the results, a validation set is utilized, captured by the same experimental setup. This validation set serves to validate the model and assess the performance of the obtained results against known values. The resulting system, comprising the field sensor and the deep learning technique, is deemed intelligent and capable of predicting the correspondence between current values, frequencies, and their respective waterfalls.

The paper outlines a novel approach to the contactless measurement of the current at industrial frequencies using a combination of magnetic field data, spectrograms, and deep learning techniques.

The advantages of the proposed approach, compared with other measurement systems, are the contactless and non-invasive way of conducting the measurements. Specifically, contact with the neither the conductor nor the electric circuit is needed. Moreover, there is no need to modify the circuit for measuring. In contrast, other measurement devices, like a shunt resistor and current transformers, need a contact with the electric circuit or a modification of it. Another advantage is the cost; the proposed method is cheap with respect to other methods, like fiber-optic current sensors. Nevertheless, the accuracy of the proposed method is mainly based on the field sensor; if high accuracy is needed, an accurate field sensor could be used instead of a less accurate one, but the costs would increase. Based on the last remark, it is worth noting that the proposed approach is flexible, because it allows for the use of different field probes, depending on the application. The drawback is that, depending on the sensor, new training of the deep learning approach is needed.

The paper is structured as follows: in Section 2.1, the measurement setup is detailed, while Section 2.2 provides an explanation of the deep learning approach, including the training procedure. Section 3 presents and discusses the results for the six considered cases, and Section 4 draws conclusions based on the findings.

## 2. Materials and Methods

The proposed method relies on the process of spectrograms (waterfalls) collected with a measurement campaign. The measured magnetic field is given by a sinusoidal current, for which intensity and frequency can vary, depending on the case. It might seem to be a case study, which could be even simulated by means of an analytical approach, i.e., by applying the Biot–Savart law. The choice to measure the field, instead of calculating it, is based on a two-fold reason: first, the measured quantity leads to a more realistic scenario because of uncertainties and noise added to the processed data by the measurement system. Moreover, it allows a more general procedure, able, in principle, to handle the field generated by conductors of complex geometry, current waveforms given by non-linear loads, and varying in time to be set up (simulating e.g., grid faults). These scenarios could not be easily treated by means of an analytical approach.

### 2.1. Data Acquisition

**Figure 1:**
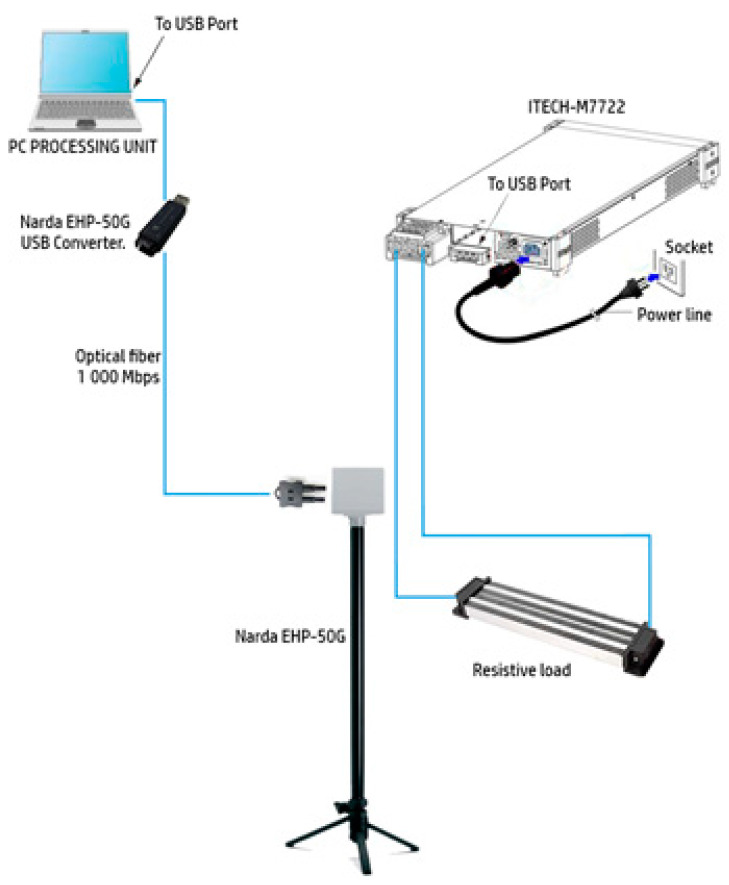
Depiction of the experiment arrangement.

The experimental arrangement is depicted in Figure 1, wherein an ITECH IT-M7722 (ITECH ELECTRONIC Co., New Taipei City, Taiwan) electronic programmable AC power supply provides power to a resistive load. The built-in power meter and arbitrary waveform generator of the ITECH IT-M7722 enable the simulation of various waveform outputs to fulfill the requirements of data collection. This instrument operates within a maximum range of 600 V and 6 A, with a maximum power output of 600 W/600 VA, and functions within the frequency range of 45–1000 Hz [14].

In the experiments, the electronic generator supplies a sinusoidal voltage at a given frequency, and the current flowing in the resistive load has approximately the same waveform since the impedance of the resistance has no relationship with the frequency (passive component). The insulation of the current-carrying wire, connecting the generator to the load, is made of plastic.

The device employed for data acquisition in this study is the Narda EHP-50G (Narda, Cisiano sul Neva, Savona, Italy, software version 2.13), a sophisticated low-frequency electric and magnetic isotropic field probe designed to facilitate advanced field analysis. This probe is specifically engineered to operate within the frequency range of 1 Hz to 400 kHz, offering a high dynamic range to capture a comprehensive spectrum of signals. The lowest field range that can be measured by this probe (the range used for the measurements in this study) is from 0.3 nT to 100 µT, the dynamic range is 110 dB, and the resolution is 0.1 nT.

One of the notable features of the Narda EHP-50G is its capability for simultaneous measurements along the *X*, *Y*, and *Z* axes, providing a comprehensive understanding of the electromagnetic field characteristics. The built-in spectrum analyzer enhances its versatility, allowing for real-time analysis and interpretation of the acquired data.

Moreover, the probe incorporates a memory function that stores frequency and level calibration tables. This feature not only ensures accuracy and precision in measurements but also streamlines the calibration process, contributing to the reliability of the acquired data.

To facilitate seamless integration into monitoring systems, the Narda EHP-50G is equipped with an internal optical repeater. This innovative component enables a secure and efficient connection to the monitoring unit, typically a personal computer (PC), through an optical fiber. The use of an optical fiber not only supports high-speed data transfer but also minimizes the impact of electromagnetic interference on the acquired signals.

In summary, the Narda EHP-50G emerges as a comprehensive and sophisticated tool for low-frequency electromagnetic field analysis, offering simultaneous measurements in multiple axes, a built-in spectrum analyzer, memory storage for calibration data, and a robust optical communication interface for efficient data transfer to monitoring units. These features collectively contribute to the precision, reliability, and versatility of the device in capturing and analyzing electromagnetic fields within the specified frequency range.

**Figure 2:**
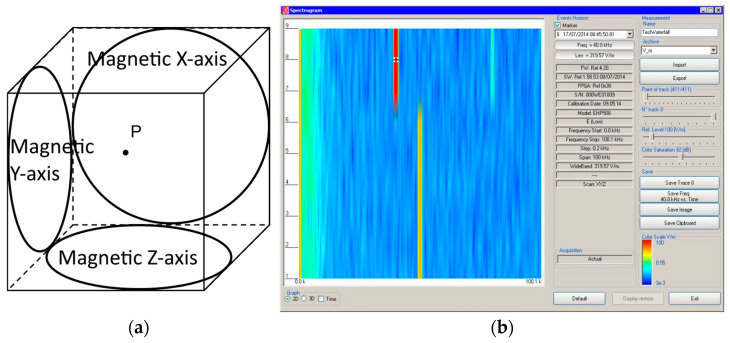
Narda EHP-50G probe: (**a**) illustration of the three orthogonal sensing loops; (**b**) probe monitoring using PC software (Spectrogram) [15].

The magnetic sensor system is composed of three magnetic loops positioned orthogonally to each other, as illustrated in Figure 2a.

Spectral analysis is monitored on the PC, as illustrated in Figure 2b.

For the sake of a comparison, the characteristics of different probes for the current measurement, available on the market, are presented in a comparative way in Table 1 [16]. The probes are based on different physical principles, and it is worth noting that the characteristics vary based on the field of application [17].

In Table 1, invasive means that a modification of the circuit is needed for utilizing the relevant sensor. Our system is contactless and does not need any circuit modification for measuring the magnetic induction field. The EHP-50G field probe is able to measure fields in the frequency range 1 Hz–400 kHz produced by currents, for which intensity varies from an ampere to thousands of amperes. The accuracy, calculated as an average over the three axes at 50 Hz and for fields up to 10 µT, is 5.6%.

The experiment consists of plotting the measured magnetic field variation with respect to either the current magnitude or frequency, which directly affects the magnetic field pattern. The variation is observed in the form of waterfall graphs that represent the intensity of the magnetic field on a frequency and time basis. In particular, the frequency is plotted along the *x*-axis, while the time is plotted along the *y*-axis of the waterfall.

The EHP-50G probe is sensitive to any electric and electronic devices that are in the vicinity of the circuit; therefore, the measures were made under strict conditions to assure the reliability of the data, i.e., all possible magnetic field sources at these frequencies were moved away from the measurement system.

The position of the Narda EHP-50G probe could affect the accuracy of the magnitude and frequency identification of the current, and hence, the authors took care to leave the probe at the same distance from the conductor during all the experimental measurement sessions. However, it is known that the magnetic field is inversely proportional to the distance from the wire, in case of infinite current-carrying wires. Under this hypothesis, it would be possible to change the distance of the probe from the wire, considering this distance as a further variable of the system.

Using Narda EHP-50G software, the full visualization of the magnetic field spectrum is possible, and the relevant waterfall is saved as a JPG image. This image is further processed and fed to the convolution neural network. The convolution neural network has the aim of understanding the patterns of the provided dataset, extract the features, and predict the intensity and frequency of the current. If the network is properly trained, a generalization is possible; a good prediction of current intensity and frequency, given the measured magnetic field, is possible even starting from previously unseen data.

### 2.2. Deep Learning Approach

#### 2.2.1. Database Creation

The collected image waterfalls are divided into 2 types of datasets that will be exploited to train the CNN and validate the obtained predictions. The dataset is composed of waterfall graphs of different frequencies with different measured currents. The rule of 80% for training and 20% for validation is used for dividing the dataset to properly train the CNN and to obtain an optimal performance.

The frequencies used during the experiments for building the current prediction dataset are 50 Hz, 150 Hz, and 250 Hz. For each given frequency, the voltage, and hence the current, varies in the range of 1–4 A.

The current values used during the experiments for building the frequency prediction dataset are 1.5 A, 2.5 A, and 4 A. For each given current value, the frequency varies in the range of 1–500 Hz.

Table 2 and Table 3 give a quantitative representation of the datasets.

For current prediction, the CNN is fed with a total dataset of 500 images as illustrated in Table 2, whereas for current dataset acquisition, the frequency is constant and the current is varied (by varying the voltage from the power supply). The probe is used to generate the waterfalls that correspond to the intensity of the magnet field created by the given current.

For instance, to form the 50 Hz current prediction dataset, we started from a fixed frequency of 50 Hz and the current is varied from 1 A to 4 A with 300 different values distributed equally in that acquisition current range. For the second subset, the frequency is fixed at 150 Hz and the current is being varied within the same range to obtain the 150 Hz current prediction dataset. Same steps are performed for 250 Hz.

The dataset for CNN for the frequency estimation is created by fixing the value of the current, as illustrated in Table 3, and varying the frequency.

For each subset of data corresponding to a specific current value, the magnetic field’s intensity remains constant, resulting in a consistent color density. The aspect under investigation involves the displacement of the vertical bar along the *x*-axis, representing the frequency. The dataset comprises 450 images for current values of 1.5 A, 2.5 A, and 4 A. To achieve a uniform distribution of frequencies within each dataset, the 1–500 Hz bandwidth is partitioned across the subset’s image count.

**Figure 3:**
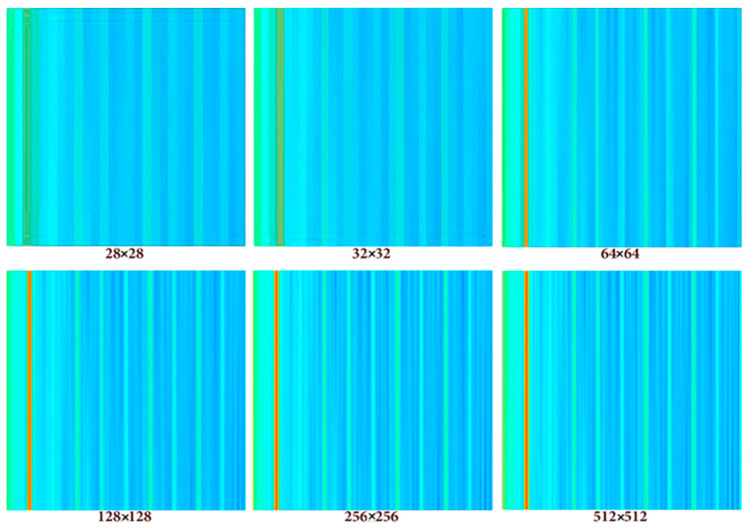
Illustrations of an image at various resolutions employed in the numerical tests.

Image resolution selection is crucial for training the CNN, and this will directly impact the performance of the model. Numerous values for resolution were tested to assess the best input size (see Figure 3). The number of pixels is decisive for the network, and the following trials show the impact of the image resolution on the behavior of the model where a larger image resolution causes a higher RMSE.

This phenomenon that opposes deep learning notions, which states that the higher resolution we feed the network the better the training, is due to the correlation between the number of samples in the database and the RMSE error. In fact, high-resolution images require deep CNN with large filters and hence more weights to optimize. This means that a large database with many samples must be used for the CNN training. In case small databases are used with high-resolution images, the RMSE error turns out to be high.

Upon closer examination, it became evident that experiments involving a 256-pixel input image led to a substantial increase in the execution duration, coupled with significant memory consumption throughout the training process. This observation highlights a crucial tradeoff between the aspiration for high accuracy and the imperative of effectively managing computational complexity.

In practical terms, the completion of a single training session with 6000 iteration steps took approximately 3.5 h. This was conducted on hardware equipped with 12 GB of RAM DDR4 and a seventh-generation processor with a clocking speed of 3.5 GHz. Notably, the total size of the processed data amounted to 64.2 GB. This context underscores the practical challenges associated with achieving high accuracy, particularly when dealing with large datasets and resource-intensive configurations. As researchers navigate these tradeoffs, optimizing both accuracy and computational efficiency remains a key consideration.

The selected resolution of 256 pixels represents a midpoint in this tradeoff, balancing the desire for accuracy with the computational resources required.

In the realm of CNN training, image resolutions typically fall within the range of 64×64 to 256×256 [18]. While it is commonly acknowledged that image resolution impacts the CNN performance, understanding and monitoring this impact are crucial. In all numerical tests, a consistent configuration and hyperparameters are maintained to systematically observe the effect of the resolution on the network.

The decision to down-sample the images to a resolution of 28×28 is motivated by the goal of minimizing the processing time and resource demands. However, it is important to acknowledge that a substantial reduction in image resolution may lead to the elimination of critical information essential for the learning process. Striking a balance among resolution, computational demands, and the preservation of meaningful data becomes a crucial consideration in optimizing the overall performance of the CNN.

**Figure 4:**
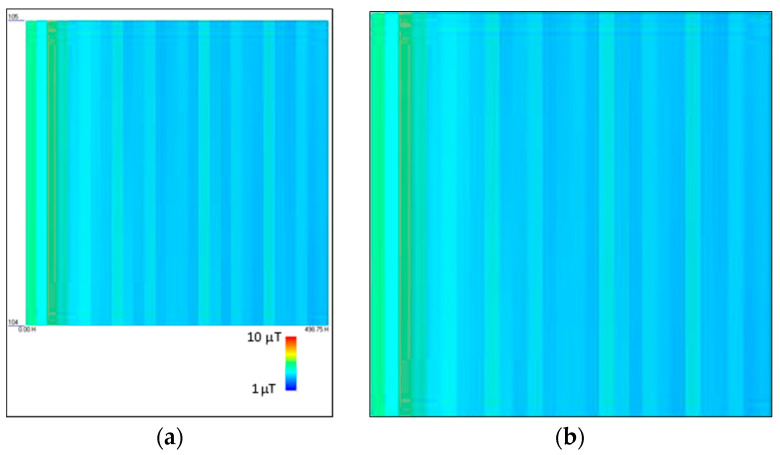
Image processing of datasets: (**a**) spectrogram before image processing; (**b**) spectrogram after image processing.

Finally, for each image, non-useful information, like, e.g., the legend and axis labels, is removed from the image, as shown in Figure 4.

#### 2.2.2. CNN Architecture and Training

CNNs are structured differently as compared to classical, fully-connected neural networks. Each layer is only connected to a small portion of neurons in the previous layer. The designed CNN is a feed-forward neural network, where the waterfall spectrograms are inserted into the input layer in the form of a single matrix with a size of 28×28×3 and the output is the predicted value of the current intensity or current frequency, depending on the case, as shown in Table 4.

**Figure 5:**
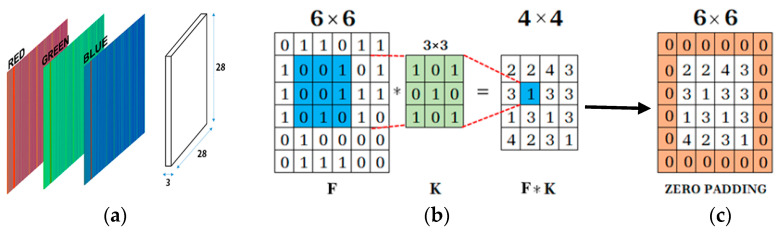
RGB to matrix form with padding: (**a**) CNN input matrix (28 × 28 × 3); (**b**) kernel filter; (**c**) zero padding added to image.

The second layer is a convolutional layer; it convolves the input by moving the filters along the input vertically and horizontally and computing the dot product of the weights and the input, as shown in Figure 5b, and then adding a bias term. The expression of the convolution is given by [19]:

(1) $$\left(f*g\right)\left[n\right]=\overset{\;m-1}{\underset{k=0}{\sum }}f\left(n-k\right)g\left(k\right)\;$$

where f(n) and g(n) are functions for the *n*th sample, m is the total number of samples, and k is the index of the new sample created through the convolution.

This layer is followed by the padding layer, which preserves the same size of the matrix to allow for a more accurate analysis of the input image.

Padding refers to the amount of pixels added to an image when it is being processed by the kernel of a CNN. For example, if the padding in a CNN is set to zero, then every pixel value that is added will be of value zero. If, however, the zero padding is set to one, there will be a one-pixel border added to the image with a pixel value of zero. Adding layers of zeros to our input images allows us to avoid the problems mentioned above. Same padding is a concept where padding layers are added such that the output image has the same dimensions as the input image, which is the desired outcome.

In this type of task, the computer program is asked to predict a numerical value to a given input. To solve this task, the learning algorithm is asked to output a function $f:R^{n}\to R$. This type of task is a regression problem [20].

The activation function chosen is ReLU (Rectified Linear Unit) due to its capability to mitigate the vanishing gradient problem, thereby enabling CNNs to learn more rapidly and achieve enhanced performance. ReLU serves as the default activation in the development of Multilayer Perceptrons (MLPs) and Convolutional Neural Networks (CNNs). It yields numerical values greater than zero and is represented by the following formula [20]:

(2) $$ReLU\left(x\right)=\{\begin{array}{c} x\;\;\;\;\;if\;x>0\\ 0\;\;\;\;\;if\;x\leq 0 \end{array}\;$$

Batch normalization is chosen as the normalization technique due to its ability to accelerate the training process and facilitate the use of higher learning rates, resulting in more stable and efficient learning. The formula for batch normalization is expressed as follows:

(3) $$z^{N}=\frac{z-m_{z}}{\sigma_{z}}$$

being z the output of the neurons, $m_{z}$ the average of the neuron output, $\sigma_{z}$ the standard deviation of the neuron output, and N the index of the neuron.

The most common problem faced when training a CNN is the overfitting/underfitting problem. Underfitting refers to a model that can neither perform well on the training data nor generalize to new data. The major causes are the high bias and low variance with the presence of noise; the size of the training dataset used has a low portion with respect to the test and validation set and due to the fact that the model is too simple. A model is said to be overfitted when the model does not make accurate predictions based on testing data. When a model gets trained with so much data, it starts learning from the noise and inaccurate data entries in our dataset, which causes false regression in our current/frequency predictions.

#### 2.2.3. Hyperparameter Tuning

In this section, a full study of the CNNs behavior is achieved by proposing several hyperparameters that control the level of accuracy of the predicted samples implicitly. The accuracy is calculated on the basis of the threshold, i.e., the tolerance. Meaning what is the amount of the allowed error to say that the data are accurate, the inference model is designed as follows:

(4) $$N_{c}=\overset{n}{\underset{i=1}{\sum }}P_{i}\{\begin{array}{c} 1,\;\;\;\;\;\;\;\;if\;E_{i}\leq Thr\\ 0,\;\;\;\;\;\;\;\;Otherwise\; \end{array}$$

(5) $$Accuracy\left(\%\right)=\frac{N_{c}}{N_{total}}\times 100$$

$N_{c}$: Number of correct predictions.

$P_{i}$: Prediction of the *i*th sample (true prediction or false prediction).

$E_{i}$: Error of the *i*th sample.

Thr: Threshold.

$N_{total}:$ Number of total predictions.

This accuracy is proportional to the variable associated with the model named Threshold that represents the admissibility of the results. The threshold value is fixed in every inference step (i.e., variable tolerance). For instance, an accuracy of 84% was obtained for the predicted values with a ±5% tolerance from the correct values.

Other parameters were introduced to investigate the best possible configurations in order to obtain the highest accuracy through a grid search. It is noticeable that the performance of the model is affected by our dataset along with the parameters of the network. The objective is finding the hyperparameters that return the highest accuracy, and the hyperparameters are as follows:

▪ Batch size.
▪ Network weight initialization.
▪ Momentum and drop period.
▪ Resolution of the input image.
▪ Learning rate.
▪ Number of epochs.

The resolution of input images was set at a fixed dimension of 28×28 pixels following a series of trials designed to encompass a range of possible resolutions, as outlined in Table 5. Hyperparameters, such as the learning rate (lr), mini-batch size (mbs), and epochs, were arbitrarily chosen without prior investigation. This approach is taken as the primary objective is to identify the optimal resolution.

The trial of the resolution 256×256 resulting from the highest error tends to be infinite. Meaning that by increasing the resolution, we are amplifying the noise until we reach the total divergence at that resolution. The resolution 28×28 is optimal, but, in order to achieve better results, other hyperparameters (learning rate Lr, Epochs, Momentum, and Drop period) need to be grid-searched. Different trials for the starting point of the learning rate have been tried; the one giving the best loss without sacrificing the speed of training is used. The speed of training is a tradeoff against the performance of the CNN.

At first, the training has started with high learning rates to form a global idea about where the optimal learning rate might be located. It was found that the optimal learning rate is in the order of magnitude of $10^{-4}$. Then, a sweep of values is performed between $10^{-3}$ and $10^{-5}$, with the other hyperparameters being fixed. When training with a smaller learning rate, at some point the value of the loss function starts decreasing in the first few iterations reaching stability rapidly with low training efficiency.

**Figure 6:**
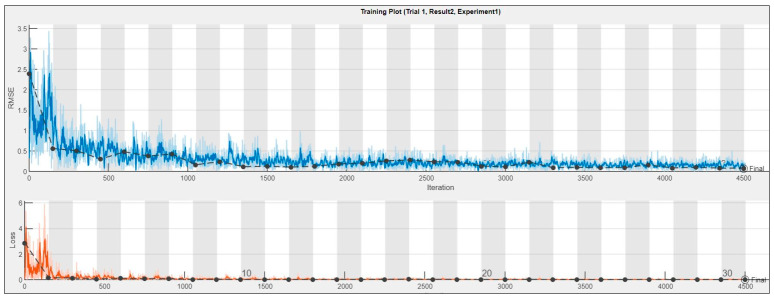
RMSE and loss curve for a given epoch.

The number of epochs decides the rate of change of the weights of the network. As the number of epochs increases, the number of weights is changed in the neural network and the decision function goes from underfitting to optimal to overfitting. Since the architecture of the designed neural network prevents overfitting, it is matter of finding the stopping point of training; after that point, certainly we will not notice any significant performance improvement, as demonstrated in Figure 6.

In the context of neural network training, the choice of the number of epochs is a critical factor that influences the learning process. A low number of epochs might lead to incomplete learning, as the model may not have sufficient iterations to adjust its weights and capture intricate patterns in the data. As illustrated in Figure 6, a fluctuating RMSE validation curve indicates instability in the training phase, emphasizing the importance of finding an appropriate epoch value.

Notably, the training loss reaching zero is not always a desirable outcome, especially in complex models or when dealing with noisy data, as it may signify overfitting. Overfitting occurs when the model memorizes the training data rather than learning general patterns, resulting in poor performance based on new, unseen data.

Grid searching is a systematic approach to finding the optimal hyperparameters, including the number of epochs. In this study, the suggested epoch interval of 10 to 40 indicates a balance between achieving satisfactory accuracy and managing computational complexity. Beyond this range, increasing the number of epochs may lead to diminishing returns and heightened computational demands without a proportional improvement in accuracy.

Table 6 provides a glimpse into different configurations and their corresponding neural network responses, highlighting the impact of epoch-tuning on the model’s performance. With a total of 940 configurations investigated, the study demonstrates a thorough exploration of the parameter space to identify the most effective training settings.

This comprehensive approach ensures that the neural network is fine-tuned for optimal performance, striking a balance between computational efficiency and accurate learning.

Gradient descent is an optimization algorithm that uses the gradient of the objective function to optimize the loss. It can be accelerated by using momentum from past updates. The momentum is unknown and has an optimal value to be searched for use; the same technique of the grid search is used. The objective consists of introducing this new hyperparameter to the configuration; the search bag is big and it takes more time. The drop period where the momentum takes effect is also as important as the momentum itself. Knowing the right time to change the momentum (decreasing the learning rate) will impact the accuracy. The momentum allows the algorithm to avoid any saddles or valleys in the training curve, meaning it can detect that training took the wrong path and it can avoid it. This hyperparameter is found by proposing a set of possible values to the previous searched configurations. The proposed drop periods are 20 and 30 since the optimal number of epochs is 40. Some of the proposed configurations are as follows.

From the analysis of Table 7, it is evident that the 7th configuration stands out as the one yielding the best results, characterized by a low validation loss. This specific configuration demonstrates an impressive accuracy of 90%. While this accuracy is commendable, it is important to note that the absolute best accuracy might not have been achieved, given the potential for further exploration.

To delve deeper into the search for optimal configurations, a grid search will be conducted with a higher number of configurations. This approach involves systematically exploring a broader range of hyperparameter combinations to identify configurations that could potentially surpass the current best accuracy.

The objective is to fine-tune model settings, expanding the exploration of the parameter space for improved performance. This iterative process aims to discover configurations that enhance accuracy and generalization capabilities.

## 3. Results

After selecting the best configurations in the previous chapter, an assessment of the CNN is made by calculating the accuracy of the prediction as it was referred to in the inference model. The accuracy is calculated using a second script to take the returned predicted values and compare them with the data provided in the validation set with the tolerance. The optimal configurations identified were incorporated into the Convolutional Neural Network (CNN), and training plots were generated. These training plots serve as valuable tools for assessing the CNN’s behavior, helping to determine whether it is prone to overfitting or underfitting. Additionally, they provide insights into the instantaneous learning process, allowing for an observation and analysis of the training loss values over time. To enhance the interpretability of the data, the predicted values were utilized to construct a scatter plot. The scatter plot serves as both a visual and statistical tool, offering a means to assess the sparsity between the predicted values. In an ideal scenario and with a well-performing learning algorithm, the sparsity would be significantly low. A low sparsity indicates that the predicted values are closely aligned with the actual data points, suggesting that the model has successfully learned and generalized from the training data.

This comprehensive approach, involving training plots and scatter plots, not only aids in evaluating the CNN’s performance but also provides a holistic understanding of its learning dynamics and predictive capabilities.

### 3.1. Current Prediction

#### 3.1.1. Current Prediction for 50 Hz

**Figure 7:**
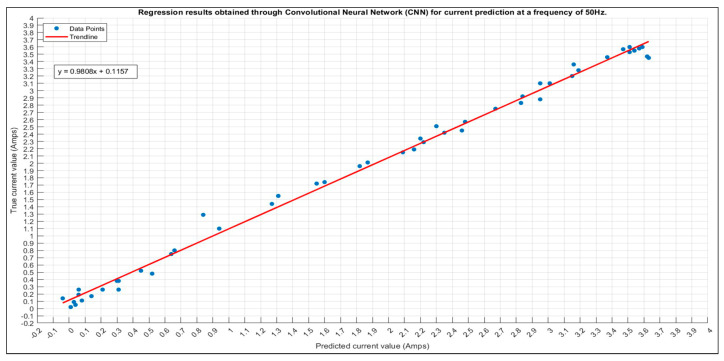
Scatter plot of the 50 Hz predicted current and the correspondent trend line.

The results obtained from the trained network with the hyperparameters in Table 8 for the 50 Hz frequency are shown in Figure 7.

**Figure 8:**
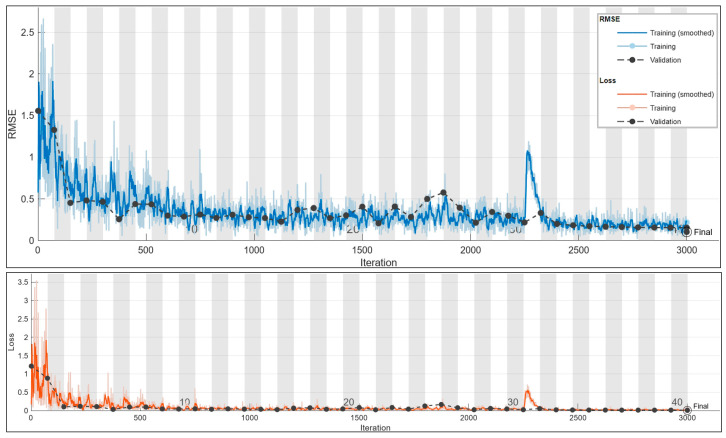
Training plot for the best configuration for the 50 Hz current prediction. Final validation loss: 0.0039. Final validation RMSE: 0.0885. Final accuracy: 98%.

The red line in the graph now serves as a trend line, representing the best fit through the actual data and indicating the overall trend observed in the predictions. Unlike the previous ideal case line, this trend line is derived from the actual data. Correct predictions falling on or near this trend line have minimal error, with the adjacent dots considered accurate as long as the predicted current error does not exceed 5% from the true value. The deviation from the trend line is evaluated to assess the accuracy of predictions, and a remarkable accuracy of 98% is achieved, with all but one predicted values falling within the tolerance band. The training plot of the CNN, consisting of 3000 iterations and shown in Figure 8, demonstrates no signs of under/overfitting, and the model attains an impressive accuracy of 98%. The best validation loss and best validation RMSE stand at 0.0039 and 0.0885, respectively.

#### 3.1.2. Current Prediction for 150 Hz

For the prediction of the 150 Hz current, a systematic approach was employed to identify the optimal configuration. A comprehensive exploration of all possible parameter values resulted in a search for the best configuration through a total of 480 trials. The most favorable outcome was achieved in trial number 298, exhibiting 80% accuracy.

In Table 9, the optimal configuration for predicting an 150 Hz current is presented, showcasing key parameter values. Our methodical parameter exploration achieves an 80% accuracy, showing the effectiveness of this approach in fine-tuning the model for optimal performance.

**Figure 9:**
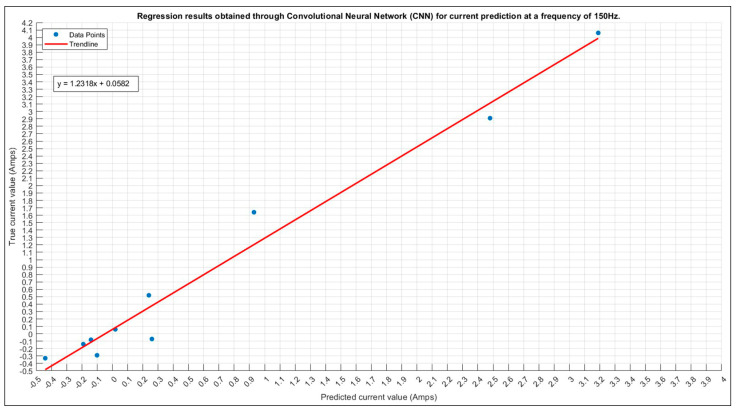
The 150 Hz current prediction scatter plot.

Employing the optimal configuration from Table 9 for predicting 150 Hz current yields commendable performance, evident in the resulting distribution. Despite minimal deviations impacting the overall accuracy, understanding and addressing these outliers is crucial. Further investigation is essential for refining the predictive model, possibly involving a reassessment of input parameters, model architecture, or training methodology. The upcoming graph (see Figure 9) compares predicted and true current values, featuring a fitted curve for enhanced visualization and insights into the model’s generalization capabilities.

**Figure 10:**
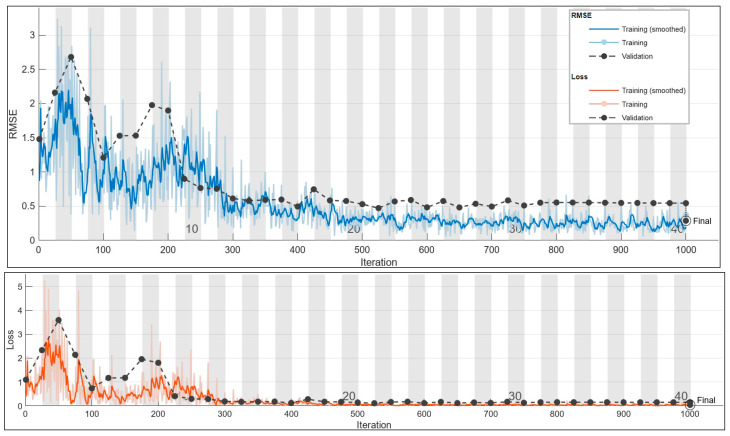
The 150 Hz current prediction training plot. Final validation loss: 0.0082. Final validation RMSE: 0.2873. Final accuracy: 80%.

In Figure 10, the training plot for the current prediction at 150 Hz is shown.

#### 3.1.3. Current Prediction for 250 Hz

**Figure 11:**
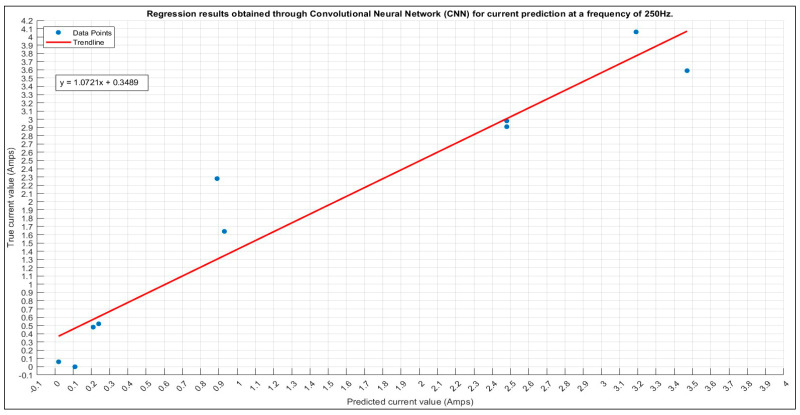
The 250 Hz current prediction scatter plot.

The ultimate prediction at 250 Hz is determined through a grid search, revealing the optimal configuration in Table 10 with the lowest error; Figure 11 presents a scatter plot illustrating the alignment between true and predicted values.

**Figure 12:**
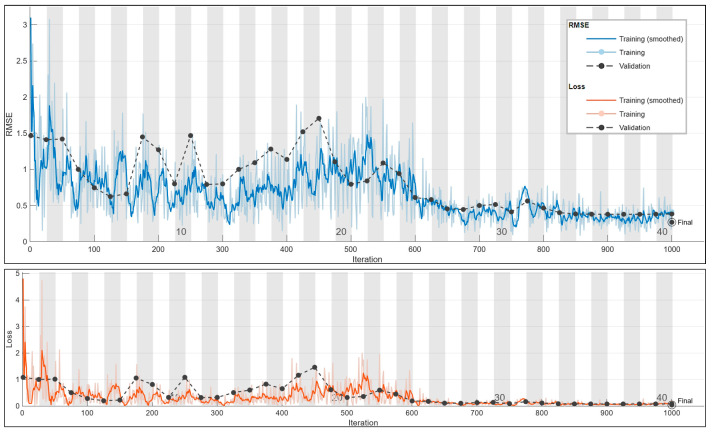
The 250 Hz current prediction training plot. Final validation loss: 0.0365. Final validation RMSE: 0.2701. Final accuracy: 70%.

In Figure 12, the training plot for the current prediction at 250 Hz is shown.

### 3.2. Frequency Prediction

#### 3.2.1. Frequency Prediction for 1.5 A

After initiating the grid search, the optimal configurations of the hyperparameters were investigated, and the best accuracy for frequency predictions is given with the hyperparameters shown in Table 11.

The CNN’s performance is assessed with the same allowed tolerance, yielding a final accuracy of 94%.

**Figure 13:**
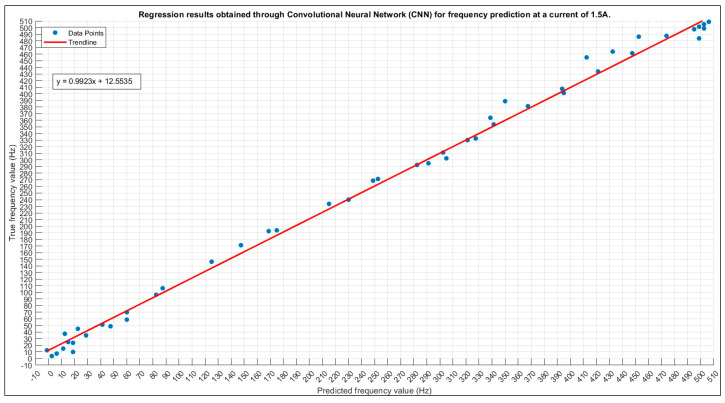
The 1.5 A frequency prediction scatter plot.

**Figure 14:**
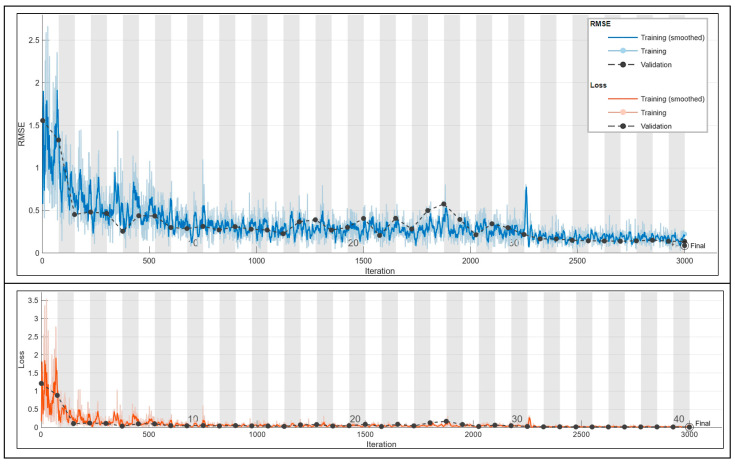
Training plot for the optimal configuration for the 1.5 A frequency prediction. Final validation Loss: 0.0055. Final validation RMSE: 0.1052. Final accuracy: 94%.

In Figure 13, the scatter plot of true versus predicted values is shown. In Figure 14, the training plot for the current prediction at 1.5 A is shown.

Initially, a marked abrupt change in the training loss is observed, but it stabilizes rapidly, attributed to the careful selection of the learning rate. The grid search methodology is utilized, allowing for the identification of optimal values without dependence on prior probabilities. This comprehensive approach considers all potential values, making it a highly accurate and potent technique. The attained accuracy stands at 94%, and further improvements can be explored by expanding the training set size and addressing magnetic field perturbations.

#### 3.2.2. Frequency Prediction for 2.5 A

An optimal configuration search has provided the hyperparameters in Table 12.

The training process exhibits a noteworthy trend with the loss values consistently reaching their highest point even after numerous iterations. Despite these challenges, the associated accuracy stands at a commendable 70%, representing the pinnacle of achievable accuracy under the current circumstances. This observation sheds light on the intricacies of the task at hand and implies potential areas for further model refinement or the Introduction of dataset augmentation techniques to enhance overall performance.

**Figure 15:**
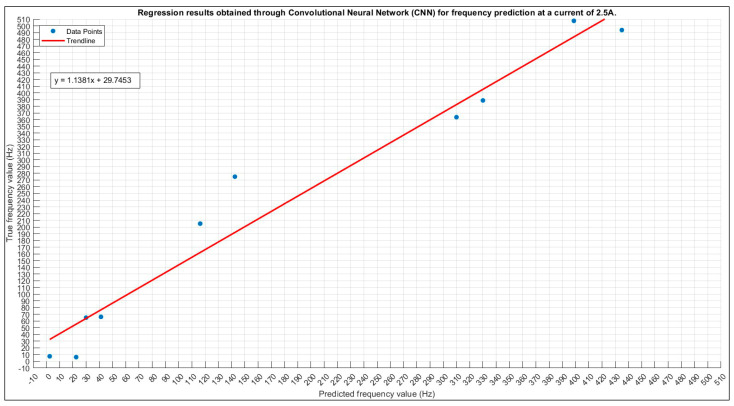
The 2.5 A frequency prediction scatter plot.

**Figure 16:**
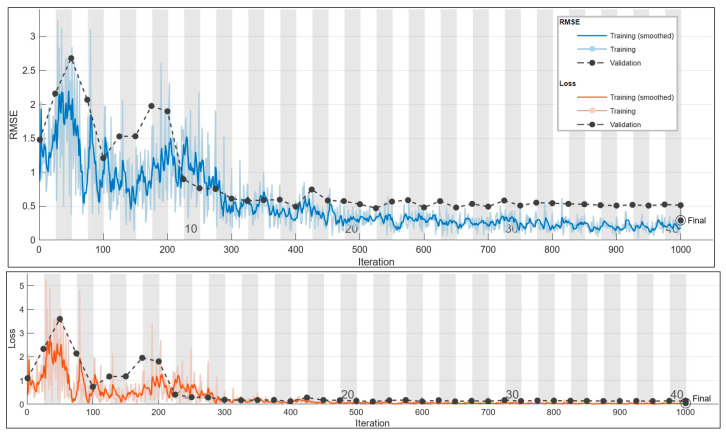
Training plot for the optimal configuration for the 2.5 A frequency prediction. Final validation loss: 0.0428. Final validation RMSE: 0.2927. Final accuracy: 70%.

In examining the results presented in Figure 15, the scatter plot vividly illustrates the correlation between true and predicted values. This visual representation is instrumental in understanding the model’s predictive capabilities and provides insights into areas of improvement. Concurrently, Figure 16 delves into the specifics of the training plot for the current prediction at 2.5 A, offering a detailed glimpse into the model’s learning trajectory and potential areas of concern or improvement.

The challenges faced during training, as evidenced by the persistent elevation in loss values, underscore the need for a nuanced approach in fine-tuning the model or exploring additional data augmentation strategies. The achieved 70% accuracy, while commendable, serves as both a benchmark and a motivation for further enhancement.

These findings collectively emphasize the dynamic nature of the machine learning process, urging a thorough investigation into model architecture, hyperparameter tuning, and potential expansion of the training dataset to unlock higher predictive capabilities and generalization. The thoughtful analysis of both quantitative and visual metrics provides a holistic perspective for refining the model’s performance in future iterations.

Utilizing the optimal configuration outlined in Table 11 for predicting the frequency with a 2.5 A current demonstrates commendable performance, as illustrated by the distribution in Figure 15. Notably, the distribution reveals that three predictions fall outside the defined tolerance range, exerting an influence on the final accuracy. This observation underscores the importance of assessing and refining the model to minimize such deviations and enhance the accuracy of frequency predictions. Further investigation and potential adjustments may be warranted to improve the model’s capability to predict frequencies within the specified tolerance range.

#### 3.2.3. Frequency Prediction for 4 A

The outcome of the exhaustive grid search process is shown in Table 13, where the optimal configuration for the model is revealed. This configuration represents the culmination of a systematic exploration across various hyperparameter values, highlighting the settings that yield the highest performance based on the specified criteria.

Achieving an impressive accuracy of 70%, the frequency prediction using the 4 A current configuration underscores the robustness and precision of the chosen setup, affirming its reliability in accurately predicting frequencies within the specific context of this study. This elevated accuracy not only serves as a quantitative metric but also attests to the effectiveness of the implemented configuration, emphasizing its suitability for this particular prediction task.

**Figure 17:**
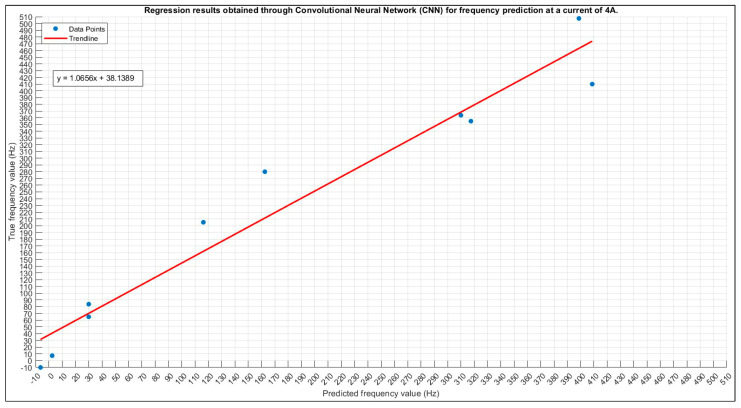
The 4 A frequency prediction scatter plot.

**Figure 18:**
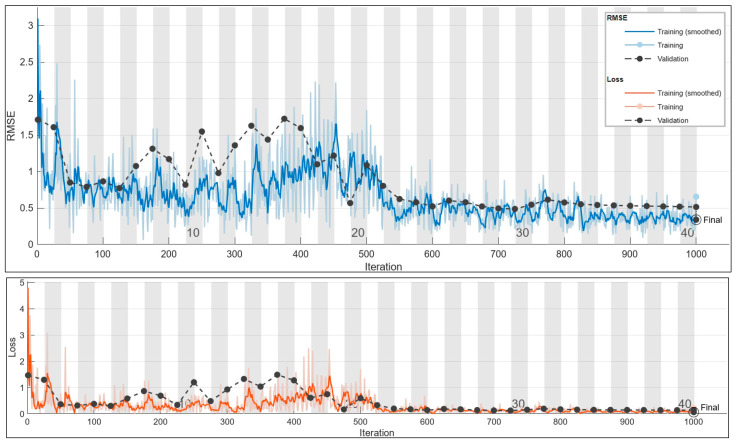
The 4 A frequency prediction training plot. Final validation loss: 0.0577. Final validation RMSE: 0.3397. Final accuracy: 70%.

The graphical representation in Figure 17, showcasing the scatter plot of true versus predicted values, visually confirms the alignment between the model’s predictions and the actual values. This visualization plays a crucial role in assessing the consistency and accuracy achieved in frequency predictions, offering a nuanced perspective based on the model’s overall performance. Additionally, Figure 18’s training plot for the 4 A current prediction provides valuable insights into the dynamics of the model’s learning process, allowing for a closer examination of convergence behavior, potential fluctuations, and overall stability during training iterations. These multifaceted evaluations, combining numerical accuracy, visual representations, and training plots, enrich the understanding of the model’s performance, laying the groundwork for informed decisions regarding potential optimizations or extensions in future research endeavors.

## 4. Conclusions

This study aimed to develop a wireless current sensing system with the primary goal of optimizing industrial practices and automating company processes. Measurements were conducted using an electromagnetic probe, specifically the Narda EHP 50-G, which translates the magnetic field into waterfall graphs. The magnetic field observed is directly proportional to the originating current.

Image-processing techniques were employed to post-process the images and create a database to train CNNs.

Achieving the highest accuracy involved employing a grid search to carefully select optimal parameters of the deep learning approach.

It is noteworthy that the proposed method is fully general and can be adapted to different setups. If a different sensor is utilized, the deep learning approach presented here could be trained with new images generated by the new sensor. The measurement range and resolution depend on the sensor used for measuring; therefore, based on the requirements of the problem, an appropriate sensor can be chosen. While the proposed method remains valid, the drawback is that new CNN training must be conducted.

The accuracy achieved with the proposed method varies from 98% for current prediction at 50 Hz to 70% at 250 Hz. This loss of performance is due to the number of samples in the database (300 samples versus 100 samples for 50 Hz and 250 Hz, respectively). The same issue occurs for the identification of the frequency: the accuracy varies from 94% for the case 1.5 A (300 samples in the database) to 70% for 4 A (50 samples in the database). The larger the number of samples, the higher the accuracy of the deep learning method.

However, the main finding of the paper is the successful demonstration that a deep learning approach can effectively identify the current’s magnitude and frequency by utilizing measurements of the magnetic induction field provided by a commercial probe. Specifically, the use of CNN, capable of processing images, lays the foundation for a system capable of handling more complex field sources, considering factors, such as geometry, waveform, and the presence of faults.

The model demonstrates the compelling synthesis of high accuracy and minimal resource requirements, positioning it favorably as a viable alternative to traditional measurement devices. Its efficient storage and retrieval of a comprehensive information database further enhance its utility, presenting diverse applications that significantly contribute to strengthening the traceability of energy consumption.

To the best knowledge of the authors, this is a novel approach and, in future work, the performance of this method, considering different conductor geometries, current waveforms, and field probes, could be investigated.

## Figures and Tables

**Table 1:** Characteristics of current sensors compared with the proposed system.

Sensor	Contactless	Invasive	Bandwidth	Accuracy	Range	Cost
Shunt resistor	N	Y	kHz–MHz	<2%	mA–A	Low
Current transformer	Y	Y	kHz–MHz	<1%	A–kA	Low
Rogowski coil	Y	N	kHz–MHz	<5%	A–MA	Medium
Field-probe based system	Y	N	1 Hz–400 kHz	5.6% *	A–kA	Medium
Fiber-optic current sensor	Y	N	kHz–MHz	<1%	kA–MA	High

* The accuracy is relevant to the field probe component of the proposed system.

**Table 2:** Dataset for current prediction.

Current Prediction Dataset		
Current Range (A)	Frequency (Hz)	Number of Images
1–4	50	300
1–4	150	100
1–4	250	100

**Table 3:** Dataset for frequency prediction.

Frequency Prediction Dataset		
Frequency Range (Hz)	Current (A)	Number of Images
1–500	1.5	300
1–500	2.5	100
1–500	4	50

**Table 4:** CNN architecture.

Layers	Layers
(1) Input layer (size 28 × 28 × 3 × n)	(10) Convolution 2D 32 filters (size 3 × 3) padding same
(2) Convolution 2D eightg filters (size 3 × 3) padding same	(11) Batch normalization
(3) Batch normalization	(12) ReLU activation function
(4) ReLU activation function	(13) Convolution 2D 16 filters (size 3 × 3) padding same
(5) Average pooling layer (size 2 × 2) stride (size 2 × 2)	(14) Batch normalization
(6) Convolution 2D 16 filters (size 3 × 3) padding same	(15) ReLU activation function
(7) Batch normalization	(16) Dropout (20% probability)
(8) ReLU activation function	(17) Fully connected layer (one output)
(9) Average pooling layer (size 2 × 2) stride (size 2 × 2)	(18) Regression layer

**Table 5:** Grid search results for optimal resolution over 4500 iterations.

Trial	Lr	mbs	Epochs	Resolution (PPI)	Accuracy (%)
1	$1\times 10^{-3}$	2	30	28×28	84
2	$1\times 10^{-3}$	2	30	32×32	82
3	$1\times 10^{-3}$	2	30	64×64	2
4	$1\times 10^{-3}$	2	30	128×128	0
5	$1\times 10^{-3}$	2	30	256×256	0
6	$1\times 10^{-3}$	2	30	512×512	0

Final validation loss: 0.0040; final validation RMSE: 0.0893; final accuracy: 84%.

**Table 6:** Epoch tuning with several configurations.

Trial	Lr	mbs	Epochs	Resolution	Training RMSE	Training Loss	Validation RMSE	Validation Loss
1	$6\times 10^{-4}$	2	30	28	0.1745	0.0152	0.0734	0.0027
2	$6\times 10^{-4}$	2	32	28	0.1140	0.0756	0.0756	0.0029
3	$6\times 10^{-4}$	2	34	28	0.1536	0.3256	0.3256	0.0530
4	$6\times 10^{-4}$	2	36	28	0.0032	0.2101	0.2101	0.0221
5	$6\times 10^{-4}$	2	38	28	0.0025	0.0783	0.0783	0.0031
6	$6\times 10^{-4}$	2	40	28	0.0224	0.1018	0.1018	0.0052

Best validation loss: 0.0027; best validation RMSE: 0.0734; best accuracy: 88%.

**Table 7:** Momentum and drop period tuning.

Trial	lr	mbs	Epochs	Resolution	Momentum	Drop Period
1	$6\times 10^{-4}$	2	40	28	0.1000	20
2	$9\times 10^{-4}$	2	40	28	0.1000	20
3	$6\times 10^{-4}$	2	40	28	0.0100	20
4	$9\times 10^{-4}$	2	40	28	0.0100	20
5	$6\times 10^{-4}$	2	40	28	0.1000	30
6	$9\times 10^{-4}$	2	40	28	0.1000	30
7	$6\times 10^{-4}$	2	40	28	0.0100	30
8	$9\times 10^{-4}$	2	40	28	0.0100	30

Final validation loss: 0.0023; final validation RMSE: 0.0685; final accuracy: 90%.

**Table 8:** Optimal hyperparameter configuration for the 50 Hz current prediction.

Trial	lr	mbs	Epochs	Resolution	Drop Period	Momentum	Validation RMSE	Validation Loss
229	$8\times 10^{-4}$	4	40	28	30	$1\times 10^{-1}$	0.0885	0.0039

**Table 9:** Optimal hyperparameter configuration for the 150 Hz current prediction.

Trial	lr	mbs	Epochs	Resolution	Validation RMSE	Validation Loss
298	$1\times 10^{-3}$	4	40	28	0.2873	0.0082

**Table 10:** Optimal hyperparameter configuration for the 250 Hz current prediction.

Trial	lr	mbs	Epochs	Resolution	Drop Period	Momentum	Validation RMSE	Validation Loss
475	$6\times 10^{-4}$	4	40	28	30	$1\times 10^{-4}$	0.2701	0.0365

**Table 11:** Optimal hyperparameter configuration for the 1.5 A frequency prediction.

Trial	lr	mbs	Epochs	Resolution	Drop Period	Momentum	Validation RMSE	Validation Loss
229	$8\times 10^{-4}$	4	40	28	30	$1\times 10^{-2}$	0.1052	0.0055

**Table 12:** Optimal hyperparameter configuration for the 2.5 A frequency prediction.

Trial	lr	mbs	Epochs	Resolution	Drop Period	Momentum	Validation RMSE	Validation Loss
348	$1\times 10^{-3}$	4	40	28	30	$1\times 10^{-1}$	0.2927	0.0428

**Table 13:** Optimal hyperparameter configuration for the 4 A frequency prediction.

Trial	lr	mbs	Epochs	Resolution	Drop Period	Momentum	Validation RMSE	Validation Loss
19	$5\times 10^{-4}$	4	40	28	30	$1\times 10^{-2}$	0.3397	0.0577

## Data Availability

Data are contained within the article.
